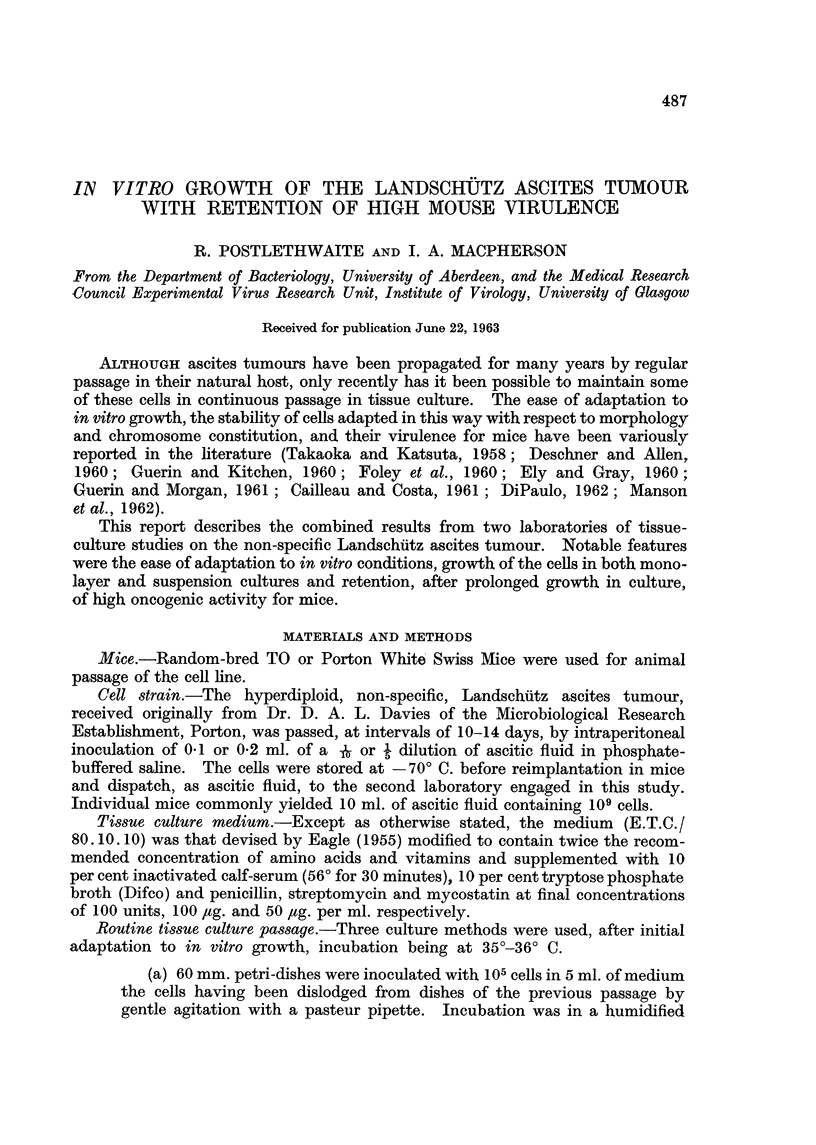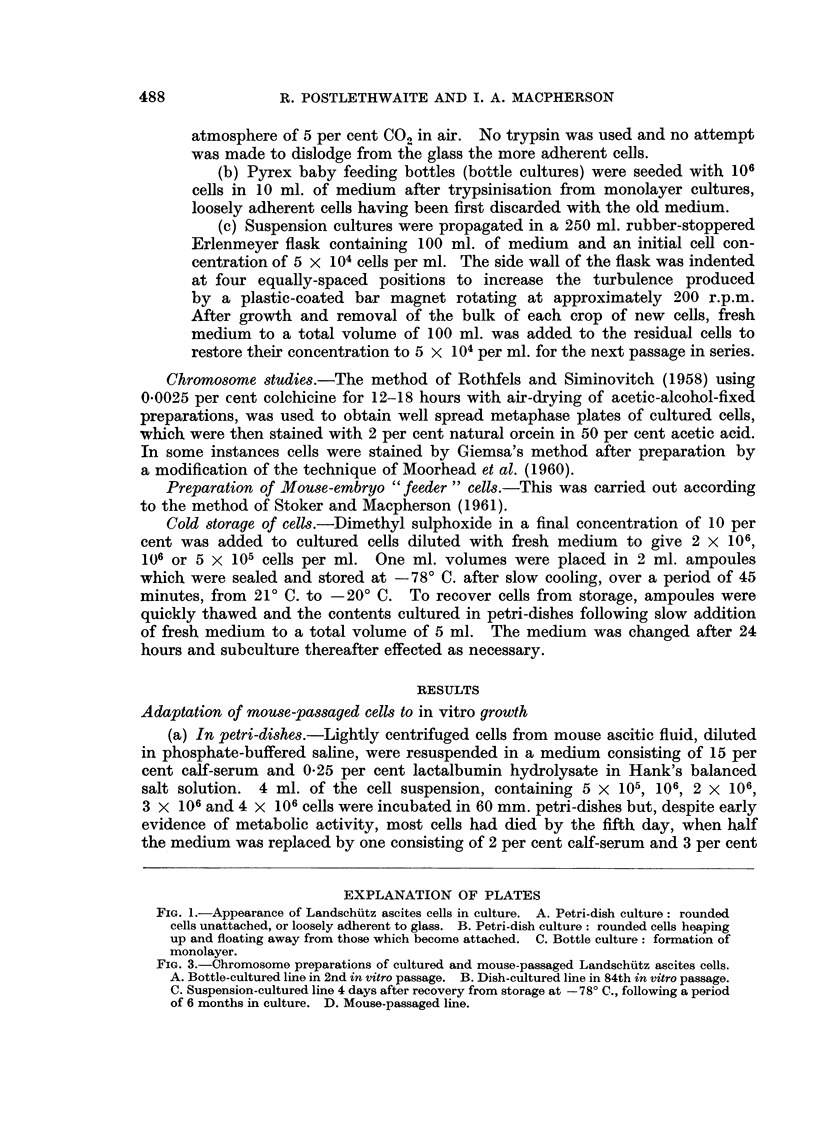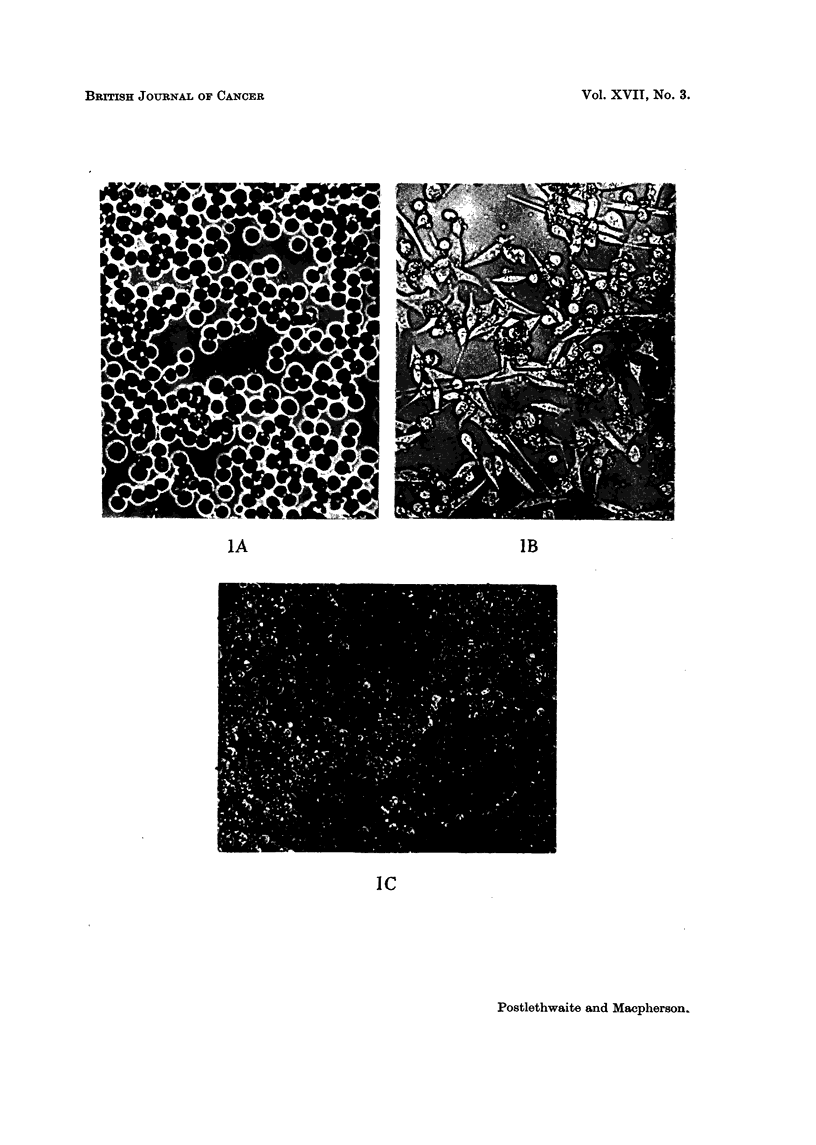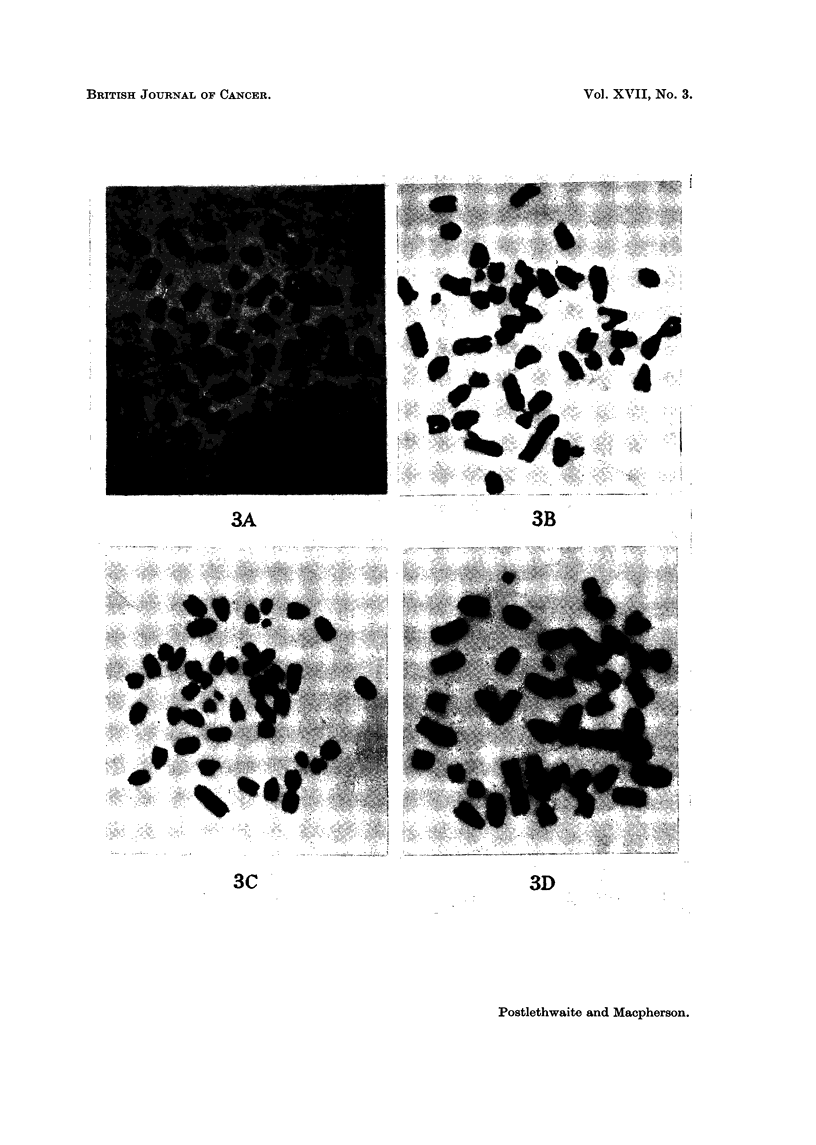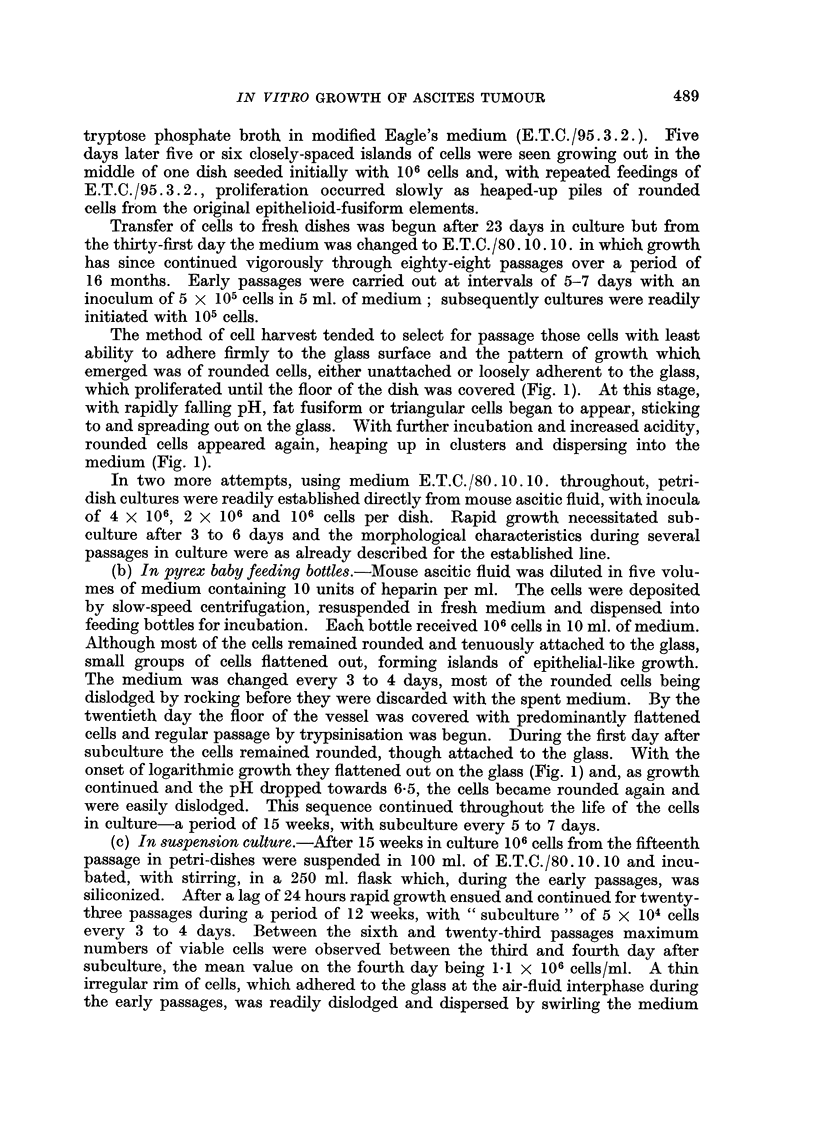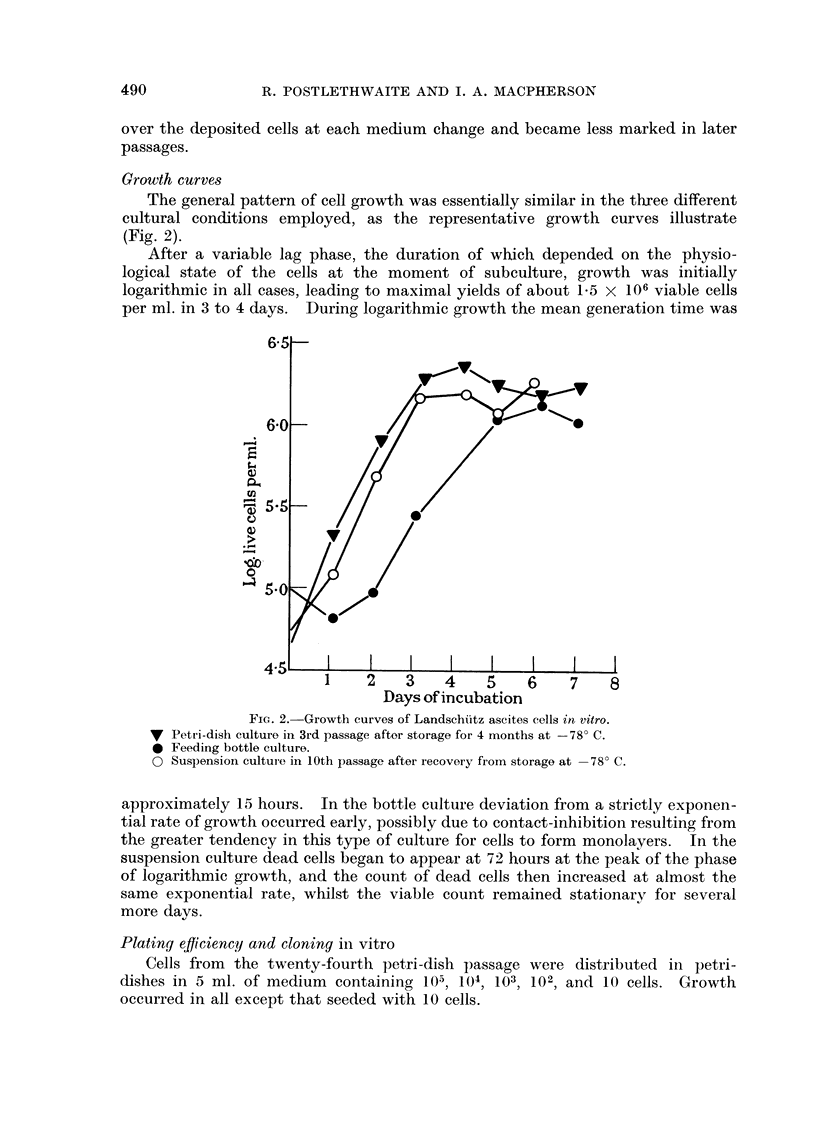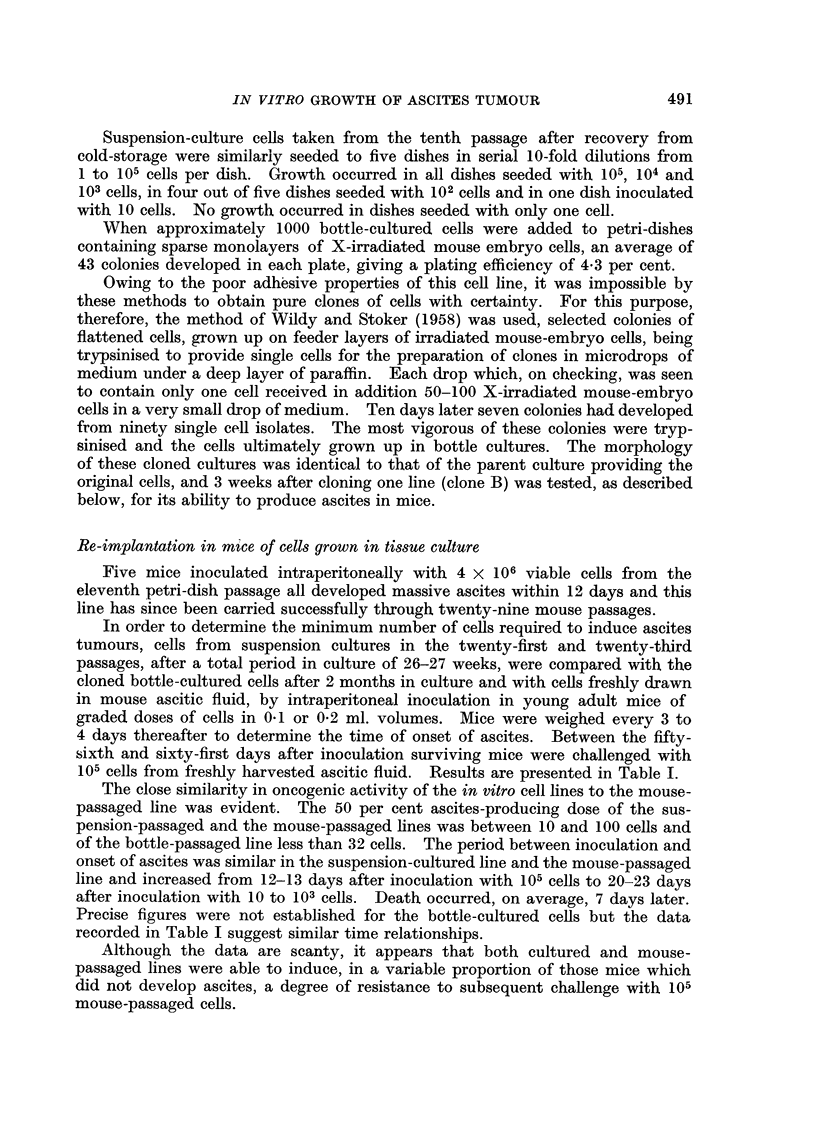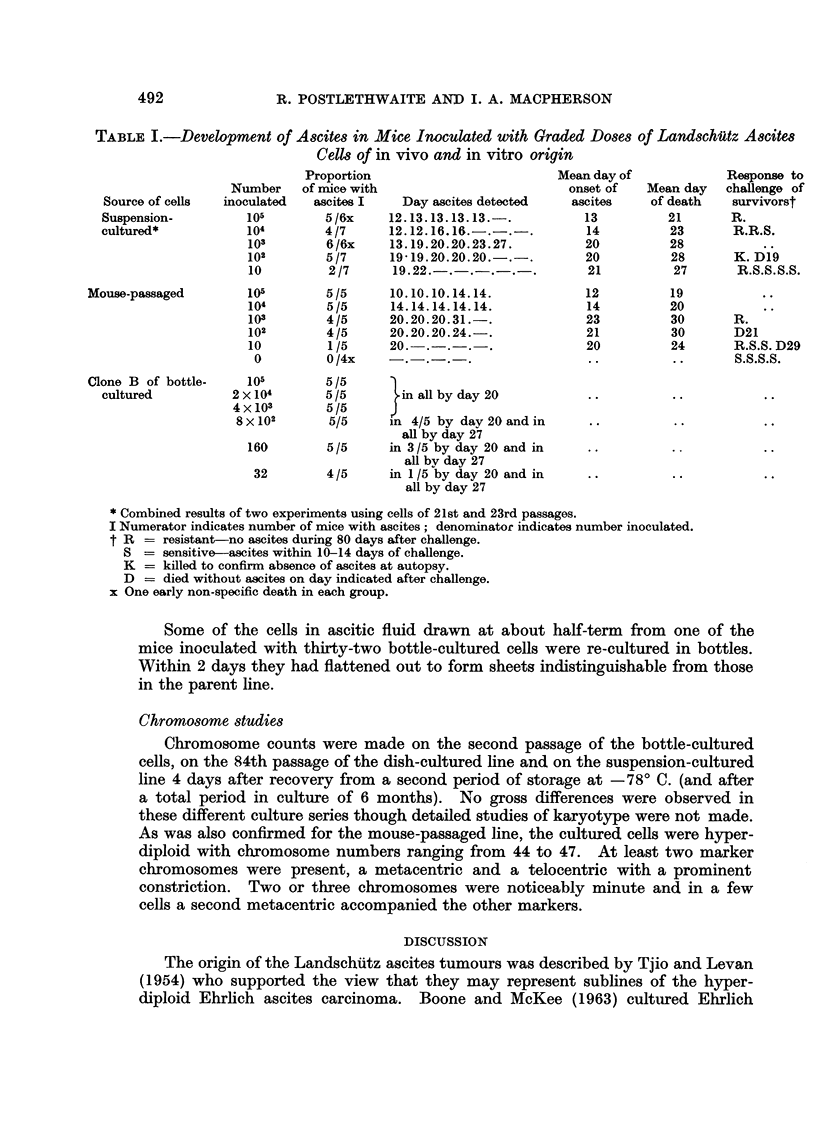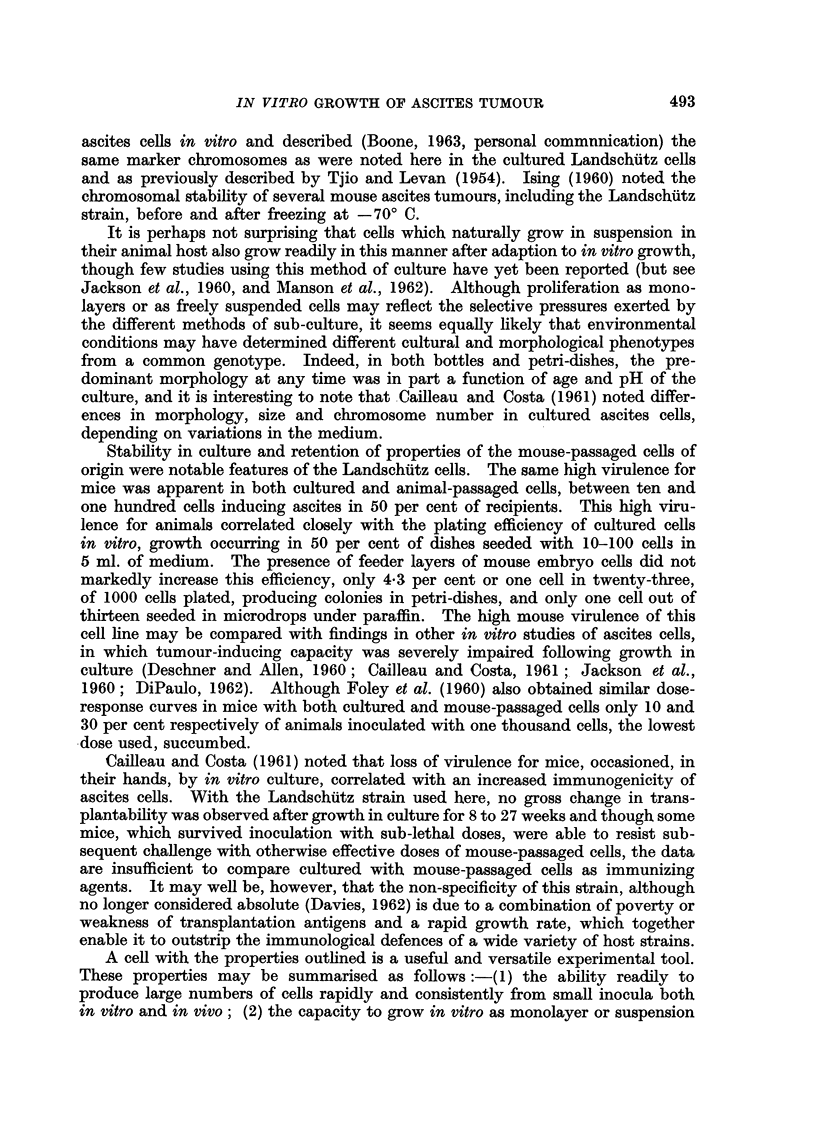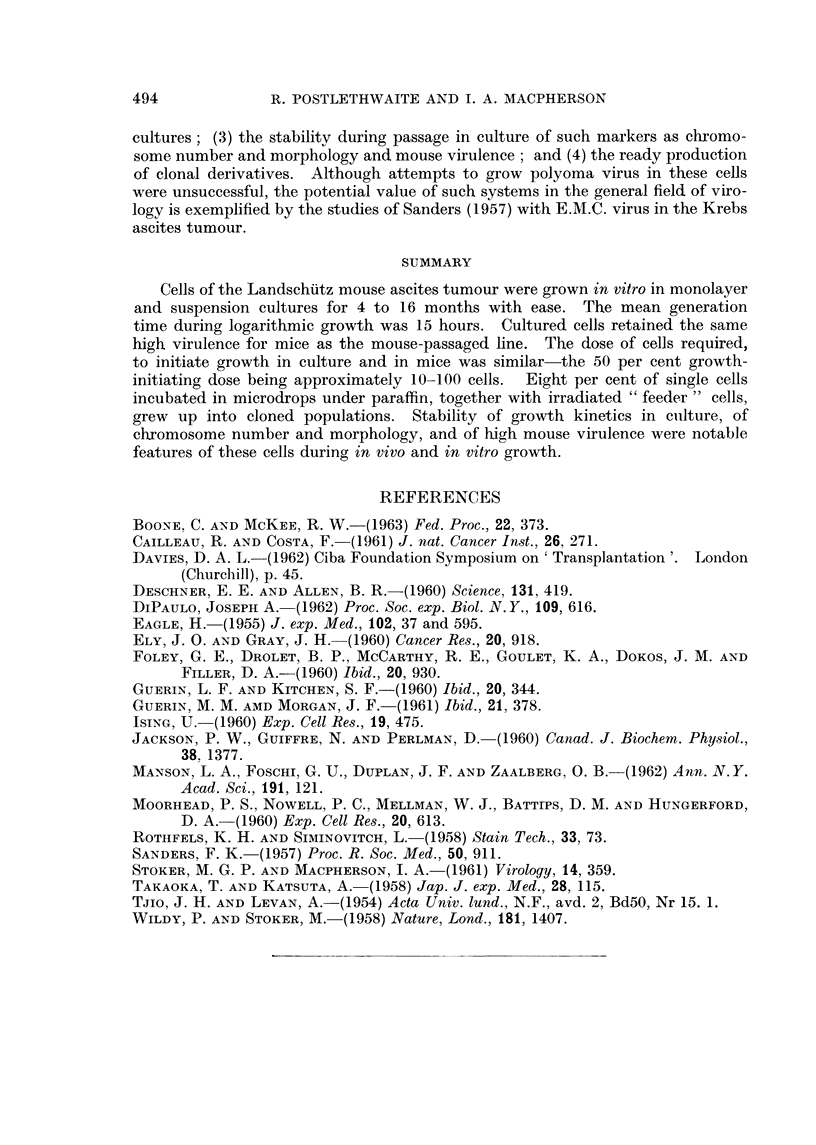# In vitro Growth of the Landschütz Ascites Tumour with Retention of High Mouse Virulence

**DOI:** 10.1038/bjc.1963.66

**Published:** 1963-09

**Authors:** R. Postlethwaite, I. A. Macpherson

## Abstract

**Images:**


### *IN VITRO* GROWTH OF THE LANDSCHÜTZ ASCITES TUMOUR WITH RETENTION OF HIGH MOUSE VIRULENCE

R. POSTLETHWAITE AND I. A. MACPHERSON

*From the Department of Bacteriology, University of Aberdeen, and the Medical Research Council Experimental Virus Research Unit, Institute of Virology, University of Glasgow*

Received for publication June 22, 1963

ALTHOUGH ascites tumours have been propagated for many years by regular passage in their natural host, only recently has it been possible to maintain some of these cells in continuous passage in tissue culture. The ease of adaptation to *in vitro* growth, the stability of cells adapted in this way with respect to morphology and chromosome constitution, and their virulence for mice have been variously reported in the literature (Takaoka and Katsuta, 1958; Deschner and Allen, 1960; Guerin and Kitchen, 1960; Foley *et al.*, 1960; Ely and Gray, 1960; Guerin and Morgan, 1961; Cailleau and Costa, 1961; DiPaulo, 1962; Manson *et al.*, 1962).

This report describes the combined results from two laboratories of tissue-culture studies on the non-specific Landschütz ascites tumour. Notable features were the ease of adaptation to *in vitro* conditions, growth of the cells in both monolayer and suspension cultures and retention, after prolonged growth in culture, of high oncogenic activity for mice.

### MATERIALS AND METHODS

*Mice.*—Random-bred TO or Porton White Swiss Mice were used for animal passage of the cell line.

*Cell strain.*—The hyperdiploid, non-specific, Landschütz ascites tumour, received originally from Dr. D. A. L. Davies of the Microbiological Research Establishment, Porton, was passed, at intervals of 10–14 days, by intraperitoneal inoculation of 0·1 or 0·2 ml. of a $\frac{1}{10}$ or $\frac{1}{5}$ dilution of ascitic fluid in phosphate-buffered saline. The cells were stored at $-70°$ C. before reimplantation in mice and dispatch, as ascitic fluid, to the second laboratory engaged in this study. Individual mice commonly yielded 10 ml. of ascitic fluid containing $10^9$ cells.

*Tissue culture medium.*—Except as otherwise stated, the medium (E.T.C./80.10.10) was that devised by Eagle (1955) modified to contain twice the recommended concentration of amino acids and vitamins and supplemented with 10 per cent inactivated calf-serum (56° for 30 minutes), 10 per cent tryptose phosphate broth (Difco) and penicillin, streptomycin and mycostatin at final concentrations of 100 units, 100 $\mu$g. and 50 $\mu$g. per ml. respectively.

*Routine tissue culture passage.*—Three culture methods were used, after initial adaptation to *in vitro* growth, incubation being at 35°–36° C.

(a) 60 mm. petri-dishes were inoculated with $10^5$ cells in 5 ml. of medium the cells having been dislodged from dishes of the previous passage by gentle agitation with a pasteur pipette. Incubation was in a humidified atmosphere of 5 per cent $CO_2$ in air. No trypsin was used and no attempt was made to dislodge from the glass the more adherent cells.

(b) Pyrex baby feeding bottles (bottle cultures) were seeded with $10^6$ cells in 10 ml. of medium after trypsinisation from monolayer cultures, loosely adherent cells having been first discarded with the old medium.

(c) Suspension cultures were propagated in a 250 ml. rubber-stoppered Erlenmeyer flask containing 100 ml. of medium and an initial cell concentration of $5 \times 10^4$ cells per ml. The side wall of the flask was indented at four equally-spaced positions to increase the turbulence produced by a plastic-coated bar magnet rotating at approximately 200 r.p.m. After growth and removal of the bulk of each crop of new cells, fresh medium to a total volume of 100 ml. was added to the residual cells to restore their concentration to $5 \times 10^4$ per ml. for the next passage in series.

*Chromosome studies.*—The method of Rothfels and Siminovitch (1958) using 0·0025 per cent colchicine for 12–18 hours with air-drying of acetic-alcohol-fixed preparations, was used to obtain well spread metaphase plates of cultured cells, which were then stained with 2 per cent natural orcein in 50 per cent acetic acid. In some instances cells were stained by Giemsa's method after preparation by a modification of the technique of Moorhead *et al.* (1960).

*Preparation of Mouse-embryo "feeder" cells.*—This was carried out according to the method of Stoker and Macpherson (1961).

*Cold storage of cells.*—Dimethyl sulphoxide in a final concentration of 10 per cent was added to cultured cells diluted with fresh medium to give $2 \times 10^6$, $10^6$ or $5 \times 10^5$ cells per ml. One ml. volumes were placed in 2 ml. ampoules which were sealed and stored at $-78°$ C. after slow cooling, over a period of 45 minutes, from 21° C. to $-20°$ C. To recover cells from storage, ampoules were quickly thawed and the contents cultured in petri-dishes following slow addition of fresh medium to a total volume of 5 ml. The medium was changed after 24 hours and subculture thereafter effected as necessary.

### RESULTS

### *Adaptation of mouse-passaged cells to* in vitro *growth*

(a) *In petri-dishes.*—Lightly centrifuged cells from mouse ascitic fluid, diluted in phosphate-buffered saline, were resuspended in a medium consisting of 15 per cent calf-serum and 0·25 per cent lactalbumin hydrolysate in Hank's balanced salt solution. 4 ml. of the cell suspension, containing $5 \times 10^5$, $10^6$, $2 \times 10^6$, $3 \times 10^6$ and $4 \times 10^6$ cells were incubated in 60 mm. petri-dishes but, despite early evidence of metabolic activity, most cells had died by the fifth day, when half the medium was replaced by one consisting of 2 per cent calf-serum and 3 per cent

---

EXPLANATION OF PLATES

FIG. 1.—Appearance of Landschütz ascites cells in culture. A. Petri-dish culture : rounded cells unattached, or loosely adherent to glass. B. Petri-dish culture : rounded cells heaping up and floating away from those which become attached. C. Bottle culture : formation of monolayer.

FIG. 3.—Chromosome preparations of cultured and mouse-passaged Landschütz ascites cells. A. Bottle-cultured line in 2nd *in vitro* passage. B. Dish-cultured line in 84th *in vitro* passage. C. Suspension-cultured line 4 days after recovery from storage at $-78°$ C., following a period of 6 months in culture. D. Mouse-passaged line.

1A

1B

1C

3A 3B 3C 3D

Postlethwaite and Macpherson.

tryptose phosphate broth in modified Eagle's medium (E.T.C./95.3.2.). Five days later five or six closely-spaced islands of cells were seen growing out in the middle of one dish seeded initially with $10^6$ cells and, with repeated feedings of E.T.C./95.3.2., proliferation occurred slowly as heaped-up piles of rounded cells from the original epithelioid-fusiform elements.

Transfer of cells to fresh dishes was begun after 23 days in culture but from the thirty-first day the medium was changed to E.T.C./80.10.10. in which growth has since continued vigorously through eighty-eight passages over a period of 16 months. Early passages were carried out at intervals of 5–7 days with an inoculum of $5 \times 10^5$ cells in 5 ml. of medium ; subsequently cultures were readily initiated with $10^5$ cells.

The method of cell harvest tended to select for passage those cells with least ability to adhere firmly to the glass surface and the pattern of growth which emerged was of rounded cells, either unattached or loosely adherent to the glass, which proliferated until the floor of the dish was covered (Fig. 1). At this stage, with rapidly falling pH, fat fusiform or triangular cells began to appear, sticking to and spreading out on the glass. With further incubation and increased acidity, rounded cells appeared again, heaping up in clusters and dispersing into the medium (Fig. 1).

In two more attempts, using medium E.T.C./80.10.10. throughout, petri-dish cultures were readily established directly from mouse ascitic fluid, with inocula of $4 \times 10^6$, $2 \times 10^6$ and $10^6$ cells per dish. Rapid growth necessitated subculture after 3 to 6 days and the morphological characteristics during several passages in culture were as already described for the established line.

(b) *In pyrex baby feeding bottles.*—Mouse ascitic fluid was diluted in five volumes of medium containing 10 units of heparin per ml. The cells were deposited by slow-speed centrifugation, resuspended in fresh medium and dispensed into feeding bottles for incubation. Each bottle received $10^6$ cells in 10 ml. of medium. Although most of the cells remained rounded and tenuously attached to the glass, small groups of cells flattened out, forming islands of epithelial-like growth. The medium was changed every 3 to 4 days, most of the rounded cells being dislodged by rocking before they were discarded with the spent medium. By the twentieth day the floor of the vessel was covered with predominantly flattened cells and regular passage by trypsinisation was begun. During the first day after subculture the cells remained rounded, though attached to the glass. With the onset of logarithmic growth they flattened out on the glass (Fig. 1) and, as growth continued and the pH dropped towards 6·5, the cells became rounded again and were easily dislodged. This sequence continued throughout the life of the cells in culture—a period of 15 weeks, with subculture every 5 to 7 days.

(c) *In suspension culture.*—After 15 weeks in culture $10^6$ cells from the fifteenth passage in petri-dishes were suspended in 100 ml. of E.T.C./80.10.10 and incubated, with stirring, in a 250 ml. flask which, during the early passages, was siliconized. After a lag of 24 hours rapid growth ensued and continued for twenty-three passages during a period of 12 weeks, with " subculture " of $5 \times 10^4$ cells every 3 to 4 days. Between the sixth and twenty-third passages maximum numbers of viable cells were observed between the third and fourth day after subculture, the mean value on the fourth day being $1{\cdot}1 \times 10^6$ cells/ml. A thin irregular rim of cells, which adhered to the glass at the air-fluid interphase during the early passages, was readily dislodged and dispersed by swirling the medium over the deposited cells at each medium change and became less marked in later passages.

*Growth curves*

The general pattern of cell growth was essentially similar in the three different cultural conditions employed, as the representative growth curves illustrate (Fig. 2).

After a variable lag phase, the duration of which depended on the physiological state of the cells at the moment of subculture, growth was initially logarithmic in all cases, leading to maximal yields of about $1{\cdot}5 \times 10^6$ viable cells per ml. in 3 to 4 days. During logarithmic growth the mean generation time was

FIG. 2.—Growth curves of Landschütz ascites cells *in vitro*.
▼ Petri-dish culture in 3rd passage after storage for 4 months at −78° C.
● Feeding bottle culture.
○ Suspension culture in 10th passage after recovery from storage at −78° C.

approximately 15 hours. In the bottle culture deviation from a strictly exponential rate of growth occurred early, possibly due to contact-inhibition resulting from the greater tendency in this type of culture for cells to form monolayers. In the suspension culture dead cells began to appear at 72 hours at the peak of the phase of logarithmic growth, and the count of dead cells then increased at almost the same exponential rate, whilst the viable count remained stationary for several more days.

*Plating efficiency and cloning* in vitro

Cells from the twenty-fourth petri-dish passage were distributed in petri-dishes in 5 ml. of medium containing $10^5$, $10^4$, $10^3$, $10^2$, and 10 cells. Growth occurred in all except that seeded with 10 cells.

Suspension-culture cells taken from the tenth passage after recovery from cold-storage were similarly seeded to five dishes in serial 10-fold dilutions from 1 to $10^5$ cells per dish. Growth occurred in all dishes seeded with $10^5$, $10^4$ and $10^3$ cells, in four out of five dishes seeded with $10^2$ cells and in one dish inoculated with 10 cells. No growth occurred in dishes seeded with only one cell.

When approximately 1000 bottle-cultured cells were added to petri-dishes containing sparse monolayers of X-irradiated mouse embryo cells, an average of 43 colonies developed in each plate, giving a plating efficiency of 4·3 per cent.

Owing to the poor adhesive properties of this cell line, it was impossible by these methods to obtain pure clones of cells with certainty. For this purpose, therefore, the method of Wildy and Stoker (1958) was used, selected colonies of flattened cells, grown up on feeder layers of irradiated mouse-embryo cells, being trypsinised to provide single cells for the preparation of clones in microdrops of medium under a deep layer of paraffin. Each drop which, on checking, was seen to contain only one cell received in addition 50–100 X-irradiated mouse-embryo cells in a very small drop of medium. Ten days later seven colonies had developed from ninety single cell isolates. The most vigorous of these colonies were trypsinised and the cells ultimately grown up in bottle cultures. The morphology of these cloned cultures was identical to that of the parent culture providing the original cells, and 3 weeks after cloning one line (clone B) was tested, as described below, for its ability to produce ascites in mice.

*Re-implantation in mice of cells grown in tissue culture*

Five mice inoculated intraperitoneally with $4 \times 10^6$ viable cells from the eleventh petri-dish passage all developed massive ascites within 12 days and this line has since been carried successfully through twenty-nine mouse passages.

In order to determine the minimum number of cells required to induce ascites tumours, cells from suspension cultures in the twenty-first and twenty-third passages, after a total period in culture of 26–27 weeks, were compared with the cloned bottle-cultured cells after 2 months in culture and with cells freshly drawn in mouse ascitic fluid, by intraperitoneal inoculation in young adult mice of graded doses of cells in 0·1 or 0·2 ml. volumes. Mice were weighed every 3 to 4 days thereafter to determine the time of onset of ascites. Between the fifty-sixth and sixty-first days after inoculation surviving mice were challenged with $10^5$ cells from freshly harvested ascitic fluid. Results are presented in Table I.

The close similarity in oncogenic activity of the *in vitro* cell lines to the mouse-passaged line was evident. The 50 per cent ascites-producing dose of the suspension-passaged and the mouse-passaged lines was between 10 and 100 cells and of the bottle-passaged line less than 32 cells. The period between inoculation and onset of ascites was similar in the suspension-cultured line and the mouse-passaged line and increased from 12–13 days after inoculation with $10^5$ cells to 20–23 days after inoculation with 10 to $10^3$ cells. Death occurred, on average, 7 days later. Precise figures were not established for the bottle-cultured cells but the data recorded in Table I suggest similar time relationships.

Although the data are scanty, it appears that both cultured and mouse-passaged lines were able to induce, in a variable proportion of those mice which did not develop ascites, a degree of resistance to subsequent challenge with $10^5$ mouse-passaged cells.

TABLE I.—*Development of Ascites in Mice Inoculated with Graded Doses of Landschütz Ascites Cells of* in vivo *and* in vitro *origin*

| Source of cells | Number inoculated | Proportion of mice with ascites I | Day ascites detected | Mean day of onset of ascites | Mean day of death | Response to challenge of survivors† |
|---|---|---|---|---|---|---|
| Suspension-cultured* | $10^5$ | 5/6x | 12.13.13.13.13.—. | 13 | 21 | R. |
| | $10^4$ | 4/7 | 12.12.16.16.—.—.—. | 14 | 23 | R.R.S. |
| | $10^3$ | 6/6x | 13.19.20.20.23.27. | 20 | 28 | .. |
| | $10^2$ | 5/7 | 19·19.20.20.20.—.—. | 20 | 28 | K. D19 |
| | 10 | 2/7 | 19.22.—.—.—.—.—. | 21 | 27 | R.S.S.S.S. |
| Mouse-passaged | $10^5$ | 5/5 | 10.10.10.14.14. | 12 | 19 | .. |
| | $10^4$ | 5/5 | 14.14.14.14.14. | 14 | 20 | .. |
| | $10^3$ | 4/5 | 20.20.20.31.—. | 23 | 30 | R. |
| | $10^2$ | 4/5 | 20.20.20.24.—. | 21 | 30 | D21 |
| | 10 | 1/5 | 20.—.—.—.—. | 20 | 24 | R.S.S. D29 |
| | 0 | 0/4x | —.—.—.—. | .. | .. | S.S.S.S. |
| Clone B of bottle-cultured | $10^5$ | 5/5 | in all by day 20 | | | |
| | $2\times10^4$ | 5/5 | in all by day 20 | .. | .. | .. |
| | $4\times10^3$ | 5/5 | in all by day 20 | | | |
| | $8\times10^2$ | 5/5 | in 4/5 by day 20 and in all by day 27 | .. | .. | .. |
| | 160 | 5/5 | in 3/5 by day 20 and in all by day 27 | .. | .. | .. |
| | 32 | 4/5 | in 1/5 by day 20 and in all by day 27 | .. | .. | .. |

* Combined results of two experiments using cells of 21st and 23rd passages.
I Numerator indicates number of mice with ascites ; denominator indicates number inoculated.
† R = resistant—no ascites during 80 days after challenge.
S = sensitive—ascites within 10–14 days of challenge.
K = killed to confirm absence of ascites at autopsy.
D = died without ascites on day indicated after challenge.
x One early non-specific death in each group.

Some of the cells in ascitic fluid drawn at about half-term from one of the mice inoculated with thirty-two bottle-cultured cells were re-cultured in bottles. Within 2 days they had flattened out to form sheets indistinguishable from those in the parent line.

### *Chromosome studies*

Chromosome counts were made on the second passage of the bottle-cultured cells, on the 84th passage of the dish-cultured line and on the suspension-cultured line 4 days after recovery from a second period of storage at −78° C. (and after a total period in culture of 6 months). No gross differences were observed in these different culture series though detailed studies of karyotype were not made. As was also confirmed for the mouse-passaged line, the cultured cells were hyper-diploid with chromosome numbers ranging from 44 to 47. At least two marker chromosomes were present, a metacentric and a telocentric with a prominent constriction. Two or three chromosomes were noticeably minute and in a few cells a second metacentric accompanied the other markers.

### DISCUSSION

The origin of the Landschütz ascites tumours was described by Tjio and Levan (1954) who supported the view that they may represent sublines of the hyper-diploid Ehrlich ascites carcinoma. Boone and McKee (1963) cultured Ehrlich ascites cells *in vitro* and described (Boone, 1963, personal commnnication) the same marker chromosomes as were noted here in the cultured Landschütz cells and as previously described by Tjio and Levan (1954). Ising (1960) noted the chromosomal stability of several mouse ascites tumours, including the Landschütz strain, before and after freezing at $-70°$ C.

It is perhaps not surprising that cells which naturally grow in suspension in their animal host also grow readily in this manner after adaption to *in vitro* growth, though few studies using this method of culture have yet been reported (but see Jackson *et al.*, 1960, and Manson *et al.*, 1962). Although proliferation as monolayers or as freely suspended cells may reflect the selective pressures exerted by the different methods of sub-culture, it seems equally likely that environmental conditions may have determined different cultural and morphological phenotypes from a common genotype. Indeed, in both bottles and petri-dishes, the predominant morphology at any time was in part a function of age and pH of the culture, and it is interesting to note that Cailleau and Costa (1961) noted differences in morphology, size and chromosome number in cultured ascites cells, depending on variations in the medium.

Stability in culture and retention of properties of the mouse-passaged cells of origin were notable features of the Landschütz cells. The same high virulence for mice was apparent in both cultured and animal-passaged cells, between ten and one hundred cells inducing ascites in 50 per cent of recipients. This high virulence for animals correlated closely with the plating efficiency of cultured cells *in vitro*, growth occurring in 50 per cent of dishes seeded with 10–100 cells in 5 ml. of medium. The presence of feeder layers of mouse embryo cells did not markedly increase this efficiency, only 4·3 per cent or one cell in twenty-three, of 1000 cells plated, producing colonies in petri-dishes, and only one cell out of thirteen seeded in microdrops under paraffin. The high mouse virulence of this cell line may be compared with findings in other *in vitro* studies of ascites cells, in which tumour-inducing capacity was severely impaired following growth in culture (Deschner and Allen, 1960; Cailleau and Costa, 1961; Jackson *et al.*, 1960; DiPaulo, 1962). Although Foley *et al.* (1960) also obtained similar dose-response curves in mice with both cultured and mouse-passaged cells only 10 and 30 per cent respectively of animals inoculated with one thousand cells, the lowest dose used, succumbed.

Cailleau and Costa (1961) noted that loss of virulence for mice, occasioned, in their hands, by *in vitro* culture, correlated with an increased immunogenicity of ascites cells. With the Landschütz strain used here, no gross change in transplantability was observed after growth in culture for 8 to 27 weeks and though some mice, which survived inoculation with sub-lethal doses, were able to resist subsequent challenge with otherwise effective doses of mouse-passaged cells, the data are insufficient to compare cultured with mouse-passaged cells as immunizing agents. It may well be, however, that the non-specificity of this strain, although no longer considered absolute (Davies, 1962) is due to a combination of poverty or weakness of transplantation antigens and a rapid growth rate, which together enable it to outstrip the immunological defences of a wide variety of host strains.

A cell with the properties outlined is a useful and versatile experimental tool. These properties may be summarised as follows:—(1) the ability readily to produce large numbers of cells rapidly and consistently from small inocula both *in vitro* and *in vivo*; (2) the capacity to grow *in vitro* as monolayer or suspension cultures ; (3) the stability during passage in culture of such markers as chromosome number and morphology and mouse virulence ; and (4) the ready production of clonal derivatives. Although attempts to grow polyoma virus in these cells were unsuccessful, the potential value of such systems in the general field of virology is exemplified by the studies of Sanders (1957) with E.M.C. virus in the Krebs ascites tumour.

SUMMARY

Cells of the Landschütz mouse ascites tumour were grown *in vitro* in monolayer and suspension cultures for 4 to 16 months with ease. The mean generation time during logarithmic growth was 15 hours. Cultured cells retained the same high virulence for mice as the mouse-passaged line. The dose of cells required, to initiate growth in culture and in mice was similar—the 50 per cent growth-initiating dose being approximately 10–100 cells. Eight per cent of single cells incubated in microdrops under paraffin, together with irradiated " feeder " cells, grew up into cloned populations. Stability of growth kinetics in culture, of chromosome number and morphology, and of high mouse virulence were notable features of these cells during *in vivo* and *in vitro* growth.

### REFERENCES

Boone, C. and McKee, R. W.—(1963) *Fed. Proc.*, **22**, 373.
Cailleau, R. and Costa, F.—(1961) *J. nat. Cancer Inst.*, **26**, 271.
Davies, D. A. L.—(1962) Ciba Foundation Symposium on ' Transplantation '. London (Churchill), p. 45.
Deschner, E. E. and Allen, B. R.—(1960) *Science*, **131**, 419.
DiPaulo, Joseph A.—(1962) *Proc. Soc. exp. Biol. N.Y.*, **109**, 616.
Eagle, H.—(1955) *J. exp. Med.*, **102**, 37 and 595.
Ely, J. O. and Gray, J. H.—(1960) *Cancer Res.*, **20**, 918.
Foley, G. E., Drolet, B. P., McCarthy, R. E., Goulet, K. A., Dokos, J. M. and Filler, D. A.—(1960) *Ibid.*, **20**, 930.
Guerin, L. F. and Kitchen, S. F.—(1960) *Ibid.*, **20**, 344.
Guerin, M. M. amd Morgan, J. F.—(1961) *Ibid.*, **21**, 378.
Ising, U.—(1960) *Exp. Cell Res.*, **19**, 475.
Jackson, P. W., Guiffre, N. and Perlman, D.—(1960) *Canad. J. Biochem. Physiol.*, **38**, 1377.
Manson, L. A., Foschi, G. U., Duplan, J. F. and Zaalberg, O. B.—(1962) *Ann. N.Y. Acad. Sci.*, **191**, 121.
Moorhead, P. S., Nowell, P. C., Mellman, W. J., Battips, D. M. and Hungerford, D. A.—(1960) *Exp. Cell Res.*, **20**, 613.
Rothfels, K. H. and Siminovitch, L.—(1958) *Stain Tech.*, **33**, 73.
Sanders, F. K.—(1957) *Proc. R. Soc. Med.*, **50**, 911.
Stoker, M. G. P. and Macpherson, I. A.—(1961) *Virology*, **14**, 359.
Takaoka, T. and Katsuta, A.—(1958) *Jap. J. exp. Med.*, **28**, 115.
Tjio, J. H. and Levan, A.—(1954) *Acta Univ. lund.*, N.F., avd. 2, Bd50, Nr 15. 1.
Wildy, P. and Stoker, M.—(1958) *Nature, Lond.*, **181**, 1407.